# REFINE (Reducing Falls in In-patient Elderly) - a randomised controlled trial

**DOI:** 10.1186/1745-6215-10-83

**Published:** 2009-09-10

**Authors:** Catherine D Vass, Opinder Sahota, Avril Drummond, Denise Kendrick, John Gladman, Tracey Sach, Mark Avis, Matthew Grainge

**Affiliations:** 1 Department of Ageing and Rehabilitation, University of Nottingham, Nottingham, NG7 2UH, UK; 2 Department of Health Care of the Older Person, Nottingham University Hospitals NHS Trust, Nottingham, NG7 2UH, UK; 3 Primary Care Research, University of Nottingham, Nottingham, NG7 2RD, UK; 4 School of Chemical Sciences and Pharmacy, University of East Anglia, Norwich, NR4 7TJ, UK; 5 School of Nursing, Midwifery and Physiotherapy, University of Nottingham, Nottingham, NG7 2UH, UK; 6 School of Community Health Sciences, University of Nottingham, Nottingham, NG7 2UH, UK

## Abstract

**Background:**

Falls in hospitals are common, resulting in injury and anxiety to patients, and large costs to NHS organisations. More than half of all in-patient falls in elderly people in acute care settings occur at the bedside, during transfers or whilst getting up to go to the toilet. In the majority of cases these falls are unwitnessed. There is insufficient evidence underpinning the effectiveness of interventions to guide clinical staff regarding the reduction of falls in the elderly inpatient. New patient monitoring technologies have the potential to offer advances in falls prevention. Bedside sensor equipment can alert staff, not in the immediate vicinity, to a potential problem and avert a fall. However no studies utilizing this assistive technology have demonstrated a significant reduction in falls rates in a randomised controlled trial setting.

**Methods/Design:**

The research design is an individual patient randomised controlled trial of bedside chair and bed pressure sensors, incorporating a radio-paging alerting mode to alert staff to patients rising from their bed or chair, across five acute elderly care wards in Nottingham University Hospitals NHS Trust. Participants will be randomised to bedside chair and bed sensors or to usual care (without the use of sensors). The primary outcome is the number of bedside in-patient falls.

**Discussion:**

The REFINE study is the first randomised controlled trial of bedside pressure sensors in elderly inpatients in an acute NHS Trust. We will assess whether falls can be successfully and cost effectively reduced using this technology, and report on its acceptability to both patients and staff.

**Trial Registration:**

**ISRCTN trial number**: ISRCTN44972300.

## Background

Falls in hospitals are common, ranging from 3 to 14 for every 1,000 bed days [1]. Over 200,000 falls were reported to the National Patient Safety Agency's (NPSA) National Reporting and Learning System (NRLS) in the 12 months from September 2005 to August 2006, with falls data being reported from 98% of organisations providing in-patient services [2]. In-patient falls result in injury and anxiety to patients and large costs to NHS organisations, including the costs of treating injuries, increased hospital stay, complaints and litigation [3-5].

Elderly in-patients are at particular risk of falling with the incidence of falls being almost triple that for community-dwelling older people [6]. Several factors contribute to this including; age, a history of falling, impaired mobility and special toileting needs. People with dementia are more likely than those without it to require hospital admission, and are at least twice as likely to fall [7-9]. Up to 30% of in-patient falls occur during the first 48 hours of the patients' admission and 75% of in-patient falls occur in the first two weeks of hospital stay [7,10,11].

Our hospital audit data are consistent with published data [2] and has shown that more than half (53%) of all in-patient falls in elderly people in acute care settings occurred at the bedside, during transfers or whilst getting up to go to the toilet. In the majority of cases these falls were unwitnessed. Where patients had been advised to call for assistance they were often reluctant to ask for help as they "did "not want to bother the nurses" or were unable to do so because of cognitive impairment.

Prevention of falls is a priority for NHS policy makers and practitioners. The Healthcare Commission has used falls prevention as a focus for inspecting compliance under patient safety [12]. Despite our increasing knowledge of the aetiology and epidemiology of falls, there is a dearth of evidence for effective falls prevention in hospitals [13-15]. There is good evidence for falls prevention in the community dwelling elderly [16-18], but these findings are unlikely to generalise to the acute hospital setting.

Advances in telecare afford innovative approaches to the reduction of falls. Assistive technology such as bed and bedside chair alarms, which alert nearby staff that a person is attempting to leave the bed or chair, are increasingly being used in the NHS [19]. However there is a lack of evidence underpinning the use of such systems. Of the few studies that have been published, none have demonstrated a significant reduction in falls rates in a randomised controlled trial setting [20,21].

The current study will address shortcomings in the evidence base. We will assess whether falls can be successfully and cost effectively reduced using pressure sensor-pager technology in a randomised controlled trial.

## Objectives

### Main research hypothesis

The hypothesis to be tested is that the use of a pressure sensor alert system, incorporating a radio-paging alerting mode to alert staff to patients rising from their bed or chair, can decrease the number of bedside falls, in older people hospitalized in an acute care setting.

### Secondary research hypotheses

1. There is a beneficial effect of pressure sensors upon health outcomes (transfer and mobility, fear of falling, quality of life, length of hospital stay and residential status on discharge) in hospitalised older people.

2. That the intervention above is cost-effective compared to usual care from a secondary care NHS perspective.

3. That there are groups of patients in whom the health or economic benefits from the intervention are greatest.

4. That the sensor system is acceptable to staff and patients and practical for use in an acute hospital setting.

## Methods/Design

**Figure 1:**
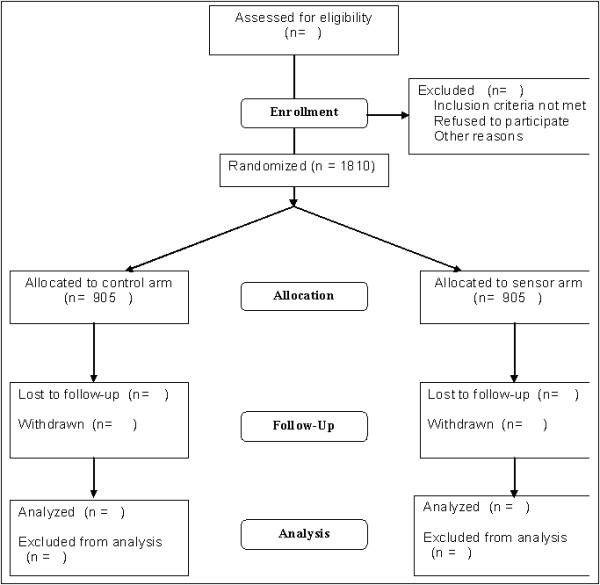
**Flow of participants through the study**.

The study is a pragmatic parallel-arm randomised controlled trial. Subjects are randomised either to receive bed and chair sensor equipment, or standard care (control arm), see Figure 1 (Flow of participants through the study).

### Setting

Five acute older persons' care wards at Nottingham University Hospitals NHS Trust (NUH).

### Participants

All patients admitted to five older persons' care wards are eligible for inclusion into the trial. Patients will be excluded from the study if they were permanently bed bound prior to admission, moribund/unconscious, or receiving end of life care on admission, or if they have previously been included in the study in an earlier admission.

### Ethics and consent

The study is executed in accordance with the principles laid down in the Helsinki Declaration [22]. This study was approved by Nottingham Research Ethics Committee 1 on the 23rd May 2008 (Reference: 07HC006; MREC: 08/H0403/40).

Written, informed consent is obtained, in accordance with International Conference on Harmonisation Guidelines for Good Clinical Practice (ICH GCP) [23]. In patients who are confused (dementia/delirium such that the subject cannot understand the nature of consenting to a research study and the study process), consent is obtained by carers or other proxies including staff, following the established framework of Berghmans, used in previous ethically approved studies in older persons with dementia [24].

### Intervention

Those participants randomised to the intervention arm of the trial will receive bedside chair and bed pressure sensors for the duration of their ward admission.

Each sensor device consists of a flexible pressure pad enveloped in thin foam encased in a durable plastic coating such that it confirms to hospital infection control policies. The bed sensor (760 mm × 250 mm × 3 mm) is placed between the bed mattress and the base of the bed in a horizontal position directly under the hip region. The chair sensor (360 mm × 175 mm × 3 mm) is positioned on top of the chair base and/or under a cushion.

The sensors detect pressure changes as the patient rises from the bed or chair, whereafter an alert is sent via radio page to a handheld pager carried by nursing staff. The pager provides the location of the call so staff can respond rapidly and ensure the patient is safe. In this way the sensor system provides an early warning to staff that the patient has left the bed or chair, and may therefore avert a potential fall.

Sensor units are leased from Tunstall Ltd. and are CE marked, complying with the regulatory authorities. Nursing staff on participating will be trained to use the sensors prior to trial recruitment. Members of the research team fit the sensors to the beds and chairs with the sensors left in place until the participant is discharged.

### Sample size

Based on data obtained from our wards during 2005, we anticipate a rate of bedside falls of 8.0 per 1,000 bed days in the control group, assuming that 53% of all falls occur at the bedside and that the average length of stay is 19 days (mean number of falls per patient = 0.15). To detect a 35% reduction in the rate of bedside falls among the intervention group, assuming 80% power, alpha = 0.05 and an over dispersion parameter of 1.5 (to allow for non-independence of falls within individuals), 905 participants are required in each arm of the trial.

### Randomisation

Subjects are allocated to the sensor or control arm using the web based randomisation service provided by the Clinical Trials Support Unit, University of Nottingham. Randomisation is determined by a computer-generated pseudo-random code using random permuted blocks of randomly varying size, created by the Nottingham Clinical Trials Unit (CTU) in accordance with their standard operating procedure (SOP) and held on a secure server, employing the Stata add-in ralloc [25]. Participants are allocated with equal probability to the sensor or control arm of the study. The randomisations are requested through a PC with internet explorer and internet access. The system is located on a dedicated secure server within the University of Nottingham. All communications between the user's PC and the server are fully encrypted (secured SSL 128 bit encrypted) and via a unique username and password.

### Blinding

In order to mitigate against nurses preferentially giving attention to patients with sensor equipment (i.e. the intervention arm) as compared to those without equipment (i.e. the control arm), we wished to find out if the nurses could be blinded to treatment allocation by the use of "dummy" (inactive) sensor equipment. To test the feasibility of this, we carried out a pilot study conducted over two days, with three 2 hour observation periods each day. Six sensors (4 active and 2 "dummy" sensors) were used with six patients, with one nurse (blinded to the status of the sensors) responding over each 2 hour period. Five nurses were assessed (the fifth nurse on the second day underwent two periods of observations). Four of the five nurses successfully identified the "dummy" versus the active sensors over the observation period. On the basis of this we concluded that it was not feasible to blind nurses to dummy verses active sensor, and therefore we decided not to use dummy sensor equipment with the control arm patients, who will be randomised to standard care alone. Any impact upon the standard care of the control group will be assessed in a qualitative sub study comprising of a non-participant observational study, and in-depth interviews with ward staff.

In order to reduce observer bias, the primary outcome measure is assessed from hospital incident reporting forms by a researcher blinded to treatment group allocation. Secondary outcome participant interviews are conducted away from the bedside so researchers remain unaware of group allocation. In instances where the researcher becomes aware of the intervention the participant has received ("unblinding"), this will be recorded.

A member of the research team, blinded to sensor allocation, enters trial outcome data. To assess the success of blinding, those measuring or entering outcome data are asked to estimate intervention or standard care for each participant, and this will be compared to actual allocation at the end of the study.

### Outcome measures

#### Definition of primary and secondary outcome measures

The primary outcome measure is the number of bedside in-patient falls per 1,000 bed days from time of randomisation until the participant is discharged from the ward.

A bedside fall is defined as an unexpected event in which the participant comes to rest on the ground, floor or lower level in the area around the bedside, with the bedside being defined as the area encompassed by the curtained area surrounding the bed. For participants in side rooms, the bedside is defined as the area of the room.

The secondary outcome measures are:

a. Number of injurious in-patient falls per 1,000 bed days, defined as falls resulting in abrasion, bruise, swelling, cut, laceration, dislocation, fracture or muscle sprain or strain.

b. Length of hospital stay.

c. Residential status on discharge.

d. Transfer/mobility score and activities of daily living, measured using the Barthel Index [26].

e. Fear of falling, measured using the Modified Falls Efficacy Scale (mFES) [27] to ascertain any differential fear of falling in the two participant groups due to an increased awareness of a potential for a fall, or a greater sense of safety arising from sensor use.

f. Health related quality of life, measured using the EUROQOL EQ-5D [28].

g. Participant and staff experience of using bed and chair sensors. This sub-study will be reported separately.

#### Ascertainment of outcomes (see Table 1 Data Collection Points)

**Table 1:** Data Collection Points

*Data collection	**Baseline**	**Discharge**
Subject demographics	**X**	
Previous falls/medical history	**X**	
Cognitive ability (30 Point MMSE)	**X**	**X**
Quality of Life (EuroQol EQ-5D)	**X**	**X**
Activities of daily living (Barthel Score)	**X**	**X**
Discharge Destination		**X**
Length of stay		**X**
Fear of Falling Questionnaire (mFES)		**X**
Total number of in-patient falls (collected at the time of the index event)		**X**

##### (a) baseline data

The baseline data collected at recruitment originates from face to face interview between researcher and patient, the patient's medical and nursing notes, and/or carer.

Baseline data includes:

a. Demographic and residential details, reason for admission, time since admission.

b. Any previous history of falls, and any resulting fractures.

c. Mobility and transfer before the illness that precipitated admission, and at the time of recruitment (measured by the Barthel ADL index).

d. 30 point Mini Mental Stare examination (MMSE).

e. Health related quality of life measured using the EuroQol EQ-5D.

##### (b) follow up data

Follow up data are collected one day prior or on the day of discharge.

Follow up data includes:

a. Falls, ascertained from hospital incident reporting forms. The use of these incident forms in NUH to record falls is mandatory and enforced by clinical governance processes. Members of the research team collect copies of hospital incident reporting forms from participating wards. Data such as fall event, time, and place, injuries sustained and subsequent actions taken are recorded. Where a medical opinion has been requested, the outcome is recorded, together with any investigations requested. In addition, aggregated, anonymzed data on falls will be collected from hospital incident reporting forms for the trial wards for the 12 month period prior to the trial commencing. The rate of falls in the period prior to the trial commencing will be compared with the control arm rate of falls during the trial to explore whether the use of sensors increases the rate of falls in the control arm.

b. Transfer/mobility and activities of daily living (Barthel ADL). This information is ascertained from the participant or carer, and/or their medical and nursing notes.

c. Quality of life (EuroQol EQ-5D) ascertained by participant interview. Where a participant is unable to participate in an interview, health related quality of life is ascertained from the carer and named nurse.

d. Fear of falling (mFES), ascertained by participant interview. Where a participant is unable to take part in an interview due to poor cognitive ability, this assessment is coded appropriately and excluded from analysis.

e. Mini Mental State Examination (MMSE), through participant interview. The baseline assessment does not account for any acute delirium secondary to the patient's condition on hospitalization, which may improve with treatment. The change in delirium may have an effect on the risk of falling.

f. Length of stay and residential status on discharge, ascertained from medical records.

Data on resource use will be collected for the economic analysis and will include the costs of investigations and interventions arising from any fall whilst in hospital and the length of stay. These data will be obtained from the medical records. Unit costs will be derived from published sources. The cost of the intervention will include rental costs, plus costs of any sensor failures and cost of installing, cleaning, and responding to sensor alarms. Nursing staff on each ward will be asked to record sensor problems and reasons for sensor removal.

Nurse time taken to respond to sensor alarms will be ascertained from an observational study to measure the content and process fidelity of the intervention. The researchers fitting the bed sensors record the length of time taken to fit sensors.

### Measuring content and process fidelity of the intervention

It is important to be able to demonstrate the extent to which the bed sensors are used in the study (content fidelity) and the way in which they were used (process fidelity). We record the number of participants who do not receive the allocated intervention and reasons for this. Nursing and research staff are asked to record sensor problems and reasons for sensor removal. In order to assess process fidelity, an observational study will be conducted to monitor nurse response rates and time taken to respond, false alarms and improper use of the sensor (e.g. not re-setting the transmitter after an alert). Staffing levels will also be recorded for observation periods to explore whether ability to respond varies with staffing levels. An observer will be provided with a pager identical to those provided to the nurses and will record each alert, the response to each alert, the time taken to respond, the time taken to attend to the patient, whether it was a false alarm or not and improper use of the sensor. Observations will be undertaken over a random sample of days and time periods (two days every 6 months), covering each of the five wards, over the study period.

### Withdrawals

Participants are free to withdraw from the trial at any stage. Participants, who decide to withdraw once recruited to the study, if allocated to a sensor, will have the sensor removed and further data collection from that point stopped. Participants, if allocated to a sensor, in whom it is deemed are at risk of harm resulting from the sensor equipment (for example in cases of confusion where the participant mis-handles the sensor equipment), will have the sensor removed and further data collection from that point stopped.

### Analysis

#### Quantitative analysis

Analyses will be undertaken on an intention to treat. Intention to treat analysis is defined in this study as an a priori plan to analyse the data according to the groups to which the subjects were randomised regardless of the treatment they received.

Fall rates for each group will be expressed as number of bedside falls per 1,000 bed days. The number of bedside falls per patient will be compared between groups using Poisson regression allowing for over dispersion, with length of stay in days as an offset. Crude models containing intervention group only will be fitted initially, whilst additional models will adjust for the effects of age, MMSE and mobility/transfer scores. Interactions between each of these three variables and treatment group with respect to our primary and secondary outcome measures will also be examined, with covariates dichotomised where necessary. For other secondary outcome measures, groups will be compared using the independent samples t-test for continuous outcomes (or Mann-Whitney U test if assumptions for using the t-test are not satisfied following appropriate transformations of the data) and the chi-square test for binary/categorical outcomes. The rate of falls in the trial wards in the 12 months prior to the trial commencing will be compared with the rate of falls in the control arm during the trial using Poisson regression, allowing for overdispersion as appropriate. A significance level of p < 0.05 will be used for all analyses.

#### Health economic analysis

A cost-effectiveness and cost-utility analysis will be performed using established methods [29]. The comparative analysis will be undertaken from a secondary care NHS perspective capturing direct secondary care health service costs and benefits over the study period. In addition to measuring the cost of the intervention, the resources consumed in the two arms of the study are likely to differ as there is the potential for differential rates of investigations and treatments, and differential lengths of stay in hospital. Resources will be valued using published unit cost data or where needed using locally derived unit costs. In this study we will use falls prevented as the effectiveness measure and change in utility as measured using the EuroQol EQ-5D as baseline and discharge [30].

An incremental economic evaluation comparing the sensor group to the standard care will be undertaken to estimate mean incremental cost-effectiveness. If one group is clearly dominant (less costly and more effective) a recommendation will be made. If non-dominance occurs (that is if costs are greater and the intervention is more effective or if the intervention is cheaper and less effective), an incremental cost-effectiveness ratio will be produced.

Probabilistic sensitivity analysis will be undertaken to test the robustness of the results. The confidence region around the incremental cost effectiveness ratio will be estimated using appropriate statistical techniques such as the non-parametric bootstrap method. This stochastic analysis will enable cost effectiveness acceptability curves and a cost effectiveness acceptability froniter to be produced [31] illustrating the uncertainty surrounding the optimal decision. Estimates of the incremental cost, incremental cost effectiveness and the uncertainty around these estimates will enable us to examine the implications of rolling out sensors nationally.

#### Project management, governance and administration

The trial is undertaken in accordance with the Standard Operating Procedures of the Clinical Trials Support Unit of the University of Nottingham and the International Conference on Harmonisation Guidelines for Good Clinical Practice in Clinical Trials [23].

The day-to-day management of the trial is the responsibility of the research lead, including research staff training and management. The study finance, documentation (such as assessment forms and operating procedures), data recording and storage is managed by the research lead, who also monitors progress in respect of project milestones (see Table 2, Project milestones for the study) and provides appropriate reports to the Trial Management Group and Trial Steering Committee. The principal investigator will instigate audits of procedures as required and takes overall responsibility for any protocol changes throughout the study.

**Table 2:** Project milestones for the study.

**Project milestones REFINE**	
2005.	"Bed Sensors Reduce In-patient Falls and Hospital Length of Stay" [32]
2005.	Health and Social Care Technology Award for the Midlands and East
2006.	Planning for a large RCT of inpatient sensors initiated
Dec 2007.	Grant awarded by the Research for Patient Benefit Programme of the National Institute for Health Research, UK (PB-PG-0107-11112)
May 2008.	Ethics approval has been obtained from the Nottingham Research Ethics Committee (reference 07HC006).
Aug 2008.	Study commencement.
Sept 2008.	Ward staff trained, Pilot of equipment and procedures
Oct 2008.	Recruitment commenced
Dec 2010.	Recruitment completed
Feb 2011.	Last participant discharged
April 2011.	Analysis completed
July 2011.	Publication

A Trial Management Group chaired by the principle investigator, comprising investigators, research staff and patient representatives, monitors all aspects of the conduct and progress of the trial, ensures that the protocol is adhered to, and will take appropriate action to safeguard participants and the quality of the trial.

A Trial Steering Committee, comprising the trial management group and three external experts (one of whom acts as chair) has been established to ensure the study governance and conduct complies with good clinical practice guidance.

A sub-group of the trial steering committee, the Data Monitoring and Ethics Committee comprising two of the external members of the steering group (including the steering group chair) and a statistician independent to the study, will receive adverse event forms electronically and will meet as required in the event of an excessive serious adverse event rate or if other information is reported to it which raises concern over the safe conduct of the trial.

#### Confidentiality and data storage

All participant questionnaires and case note abstraction forms are stored in locked cabinets, identified by a unique participant identifier. Consent forms and other documents including the participant's name are stored separately from questionnaires and other trial documents in locked cabinets.

All computer databases include the unique participant identifier and not the name and address of the participant. The database is stored on a secure dedicated clinical trials server at the University of Nottingham. The server itself is located within in a locked server room, with restricted key access to authorised individuals. Users log into the database system by entering their username and password via a secure encrypted connection (https protocol/128 bit SSL encryption). The database automatically maintains an audit trail, which records all activity. Data will be stored for 10 years.

## Discussion

The REFINE study proposed is the largest single intervention in inpatient falls, in the UK, as far as the researchers are aware. This innovative study is among the first randomised controlled trials to examine the effectiveness and cost-utility of a bedside sensor-paging system. It will respond to the dearth of randomised trials to investigate the impact of telecare upon falls in the elderly. Moreover it will be conducted with a large number of elderly in-patients in the challenging environment of a busy acute NHS Trust. The results will establish whether this monitoring system is a cost effective intervention that patients, acute trusts and other in-patient institutes can utilize and benefit from.

### Publications

All investigators will contribute towards drafting the paper reporting the main trial findings and all investigators will be named authors on that paper, providing they fulfil the Vancouver criteria for authorship. Investigators wishing to analyze and report other findings from the study can do so on the agreement of the other investigators, and the study team will agree authorship for these papers, subject to the Vancouver criteria for authorship.

## Competing interests

The authors declare that they have no competing interests.

## Authors' contributions

CDV: research lead, wrote the draft of this manuscript and obtained additional trial funding.

OS: principle investigator, conceived the project, co-led the design and co-ordination of the trial and obtained the main trial funding.

AD, DK: co-led the design of the trial and are grant holders.

TS: provided health economic advice and wrote the relevant sections of the protocol.

JG, MA: improved on the study design and are grant holders.

MG: provided statistical advice and wrote the relevant sections of the protocol.

All authors participated in the trial design, and commented, read and approved the study protocol.
